# Low‐molecular weight oligosaccharides from gum tragacanth (*Astragalus gossypinus*) ameliorate nonalcoholic fatty liver disease (NAFLD) in Wistar male rats

**DOI:** 10.1002/fsn3.3112

**Published:** 2022-10-21

**Authors:** Zahra Hossein Zadeh, Ebrahim H. Najdegerami, Mehdi Niko, Vahid Nejati, Hassan Ahmadi Gavlighi

**Affiliations:** ^1^ Department of Biology, Faculty of Science Urmia University Urmia Iran; ^2^ Department of Pathobiology and Quality Control, Artemia & Aquaculture Research Institute Urmia University Urmia Iran; ^3^ Department of Food Science and Technology, Faculty of Agriculture Tarbiat Modares University Tehran Iran

**Keywords:** autophagy, fatty liver, glycemia, gum tragacanth, oligosaccharides, oxidative stress

## Abstract

Non‐alcoholic fatty liver disease (NAFLD) is the most common liver disease affecting 25% of the world's population. The effects of oligosaccharides from Gum tragacanth (*Astragalus gossypinus*) (GT) on oxidative stress, glucose metabolism, and expression of autophagy genes were investigated in induced non‐alcoholic fatty liver. Twenty‐four male healthy rats were divided into four groups, Control; high‐fat diet, high‐fat diet + 100 mg GT oligosaccharides/kg body weight, high‐fat diet + 200 mg GT oligosaccharides/kg body weight and fed with the trial diets for 70 days. At the end of the experiment, the results indicated that GT oligosaccharides affected the weight gain and liver weight in NAFLD‐induced rats. In addition, the results showed that the use of GT oligosaccharides significantly decreased oxidative stress, liver injury, and hyperglycemia (*p* < .05) and upregulated the expression of autophagy genes in NAFLD‐induced rats.

**Practical applications:**

Overall, the results of the current study demonstrated that the use of GT oligosaccharides obtained from Gum tragacanth (*Astragalus gossypinus*) showed significant antioxidant properties and hypoglycemia in NAFLD induced rats and could be used as a useful nutritional strategy for the prevention and treatment of NAFLD.

## Graphical abstract

The current study results demonstrate that Gum tragacanth oligosaccharides show significant antioxidant properties and hypoglycemia effect in NAFLD‐induced rats and could be used as a novel nutritional strategy for the prevention and treatment of NAFLD.

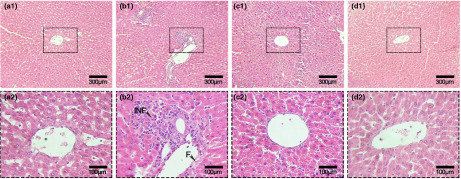

## 1 INTRODUCTION

The World Health Organization (WHO) reports the prevalence of non‐alcoholic fatty liver diseases (NAFLD) in 25% of the world's population. It should be noted that NAFLD is mainly asymptomatic and the real prevalence may be higher than that provided officially. NAFLD is characterized by an increase in the content of triglycerides and intrahepatic free fatty acids without alcohol consumption (Fabbrini et al., 2010). The progress of NAFLD can result in non‐alcoholic steatohepatitis (NASH), cirrhosis, and hepatocellular carcinoma (Bellentani, 2017) which are major mortality causes. There is no standard treatment for NAFLD (Katsiki et al., 2016); however, a few therapies like physical activity, lipid‐lowering medications, insulin sensitizers, and Pentox‐Ifyllin have been proposed to heal NAFLD (Chappuis et al., 2017; Qian et al., 2019). Despite positive effects of lifestyle management on NAFLD, the long use of chemical medicines and vitamins has raised concerns associated with side effects and have led researchers to find therapeutic approaches from natural sources to ameliorate NAFLD.

Recently, products with natural origin have attracted extensive attention due to potentially antioxidant properties and their safety (Fu et al., 2016). Among the natural products, polysaccharides are widely used in the food thickener, emulsifier, cosmetic, and pharmaceutical industries (Cho & Leung, 2007; Fu et al., 2014; Whistler, 1993). Gum tragacanth (*Astragalus gossypinus*) is a polysaccharide extracted from the *A. gossypinus* stem. The gum tragacanth is a source of several active components such as glycosides, alkaloids, amino acids, flavonoids, trace minerals, saponins, and sugars (Hao et al., 2021). The beneficial effects of Astragalus root (*Radix astragali*) polysaccharides on liver protection, neuroprotection, oxidative stress, immunity, and inflammation have been confirmed (Jiang et al., 2010; Tiippana et al., 2016). GT polysaccharides reduced the amount of reactive oxygen species (ROS) associated with lipid peroxidation and protein oxidation in the liver (Begriche et al., 2006). However, polysaccharides possess high molecular weight (MW), viscosity, and are difficult to enter the bloodstream through the intestine.

Degradation of polysaccharides by microbial enzymes and conversion to oligosaccharides with lower MW would increase their bioavailability (Zhu et al., 2017) and might exhibit some novel physiological properties because of their different molecular structures and physicochemical properties (de Moura et al., 2015; Roberfroid et al., 2010). The results have shown that oligosaccharides especially at low dose easily enter the bloodstream due to their low MW and show antioxidant and immunomodulatory effects (Chappuis et al., 2017; Morel et al., 2015; Nauta & Garssen, 2013). The literature related the effects of other oligosaccharides like chitosan and galacto‐oligosaccharides have highlighted in anti‐obesity, inflammatory response, reducing the level of blood cholesterol, free radical scavenging and alleviating certain acute or chronic diseases in animal models (Dai et al., 2014; Gosling et al., 2010), whereas the liver‐protective effect of GT oligosaccharides for NAFLD remains obscure.

Based on the aforementioned explanations and the roles of natural products in suppression of oxidative stress, the present study was designed to investigate the effects of GT oligosaccharides on oxidative status, glucose regulation, liver histopathology, and expression of genes related to autophagy in induced NAFLD male rats.

## 2 MATERIALS AND METHODS

### 2.1 Preparation of GT oligosaccharides

**FIGURE 1:**
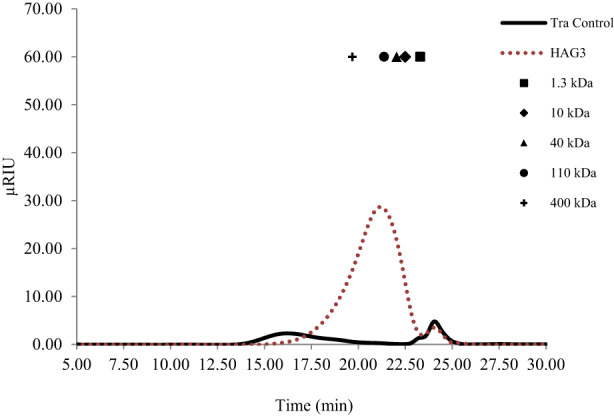
Molecular size profiles of un‐hydrolyzed Tragacanth gum (*Astragalus gossypinus*) sample (Tra control) and hydrolysate (HAG3) obtained after enzymatic modification with the Pectinex enzyme. Pharmacia dextran (T10, T40, and T110 kDa) and pullulan (1.3 and 400 kDa) (symbol arrows) were used as molecular weight standards to determine the average molecular weight. The HAG3 fraction, which belongs to the high molecular weight fraction, has an average size of around 110 kDa. Accordingly, the HAG3 fraction contains branched polysaccharides with degrees of polymerization (DP) of around 600–650 monosaccharides.

GT exuded from *A. gossypinus* species was provided from a local herbal store (Shahrekord, Iran). The flakes were ground using a coffee mill and sieved (40‐mesh size). Then, GT solution (10 g/L) was prepared by gently mixing 10 g of GT powder in 1 L Milli‐Q water for 2 h at room temperature. The final solution (pH = 5.5–6) of GT was heated to 50°C. Enzymatic digestion of GT was performed with Pectinex Ultra Color enzyme (equal to the addition of 25,000 PECTU/L) at a concentration of 2.5 ml/L on GT solution at a temperature of 50°C, for 2 h in a water bath, until the viscosity of the solution reduced from 1.2 to roughly 0.5 Pa s. The enzymatically treated GT solution was centrifuged at 5000 *g* for 10 min after enzyme deactivation (90°C for 10 min). Finally, supernatant was lyophilized and kept in a sealed container at −20°C until further experiment (Gavlighi et al., 2013). The results of analyses of lyophilized supernatant have been presented in Figure 1.

### 2.2 Animal experiments

The animal trial was performed at the Department of Biology (Urmia University, Iran) and all animal experimental protocols were approved by the Animal Ethics Committee of Urmia University (N: IR‐UU‐AEC‐3/1766/SC). A total of 24 male Wistar rats (10 to 11 weeks old, initial weight: 230.1 ± 22 g) were prepared from the animal house of the Department of Biology (Urmia University, Iran). The rats were kept in standard fiberglass cages (*n* = 6 rats/cage) and maintained in a room on a 12‐h light, humidity (50%) and temperature (25 ± 2 ^ͦ^ C). After 2 week adaptation with ad libitum access to standard diet (Javaneh khorasan co., Iran) and water, the rats were divided into four experimental groups:

1. Control (standard diet), and received by gavage 4 ml distilled water
2. High‐fat diet (HFD), Rats fed on HFD and received in addition daily by gavage 4 ml distilled water
3. HFD + O100 group: Rats fed on HFD and received in addition daily 100 mg oligosaccharide/kg body weight by gavage in a volume of 4 ml distilled water
4. HFD + O200 group: Rats fed on HFD and received in addition daily 200 mg oligosaccharide/kg body weight by gavage in a volume of 4 ml distilled water

To prepare the high‐fat diet, 5% fructose and 10% animal fat were added to 85% of the standard diets (Nasri et al., 2015) (Table 1). After 70 days experimental period, the rats were weighted and scarified by high dose of sodium Pentobarbitone (90 mg/kg). The blood was drawn from heart with 5 mm syringes impregnated with heparin anticoagulant and all internal organs such as brain, kidney and liver were also sampled. Plasma was separated after centrifugation (3500 rpm, 10 min) and stored at −20°C to measure inflammatory cytokines (TNF‐α, and IL6) and plasma biochemical indices. The rats' liver was divided into two parts after weighing and a portion was fixed in 10% formalin for histological examinations and the rest was kept in −20 to investigate liver enzymes, oxidative stress, and expression of autophagy genes.

**TABLE 1:** Composition of standard and high‐fat diets in the experiment

	Control	HFD
Protein	22	19.2
Lipid	3.5	11.8
Carbohydrates	50.4	43.2
Fructose	0	5
Caloric value (kcal/kg)	2850	3850

### 2.3 Fasting blood glucose, glucose tolerance, insulin, and HOMA‐IR

The OGTT was performed on the rats at 8 weeks, after 12‐h fasting but free access to water. Following overnight fasting, these rats were tested for an oral glucose tolerance test (OGTT). Blood samples were drawn from the tail vein and fasting glucose was measured by using a commercial kit (Darman Faraz kave, Esfahan, Iran). The glucose solution (2 g/kg rat weight) was administered orally to the starved rats and glucose levels in the tail vein were taken at 30, 60, 90, and 120 min using a glucometer (EmpErOr, Hsinchu city, Taiwan). Also, plasma insulin concentration was evaluated using a kit by Zelbio Co. (Berlin, Germany) based on the ELISA technique. The following formula was used to calculate HOMA‐IR: Insulin (μIU/ml) × fasting glucose (μmol/L)/22.5 (Matthews et al., 1985).

### 2.4 Measuring of liver function markers and biochemical indices in plasma

Biochemical indices and liver markers such as such as alkaline phosphatase (ALP), amylase, alanine aminotransferase (ALT), and aspartate aminotransferase (AST) activities were evaluated in plasma by commercial kits (Darman Faraz Kaveh, Isfahan, Iran) following the company standard protocols. Total triglyceride, cholesterol (TC), low‐density lipoprotein (LDL) and high‐density lipoprotein (HDL) concentration were measured in plasma by using commercial kits of Darman Faraz Kaveh (Isfahan, Iran) and the company prescribed protocol was followed. Cytokines (IL‐6 and TNF‐ɑ) in the plasma were evaluated using Zelbio kits (Berlin, Germany) according to the manufacturer's instructions.

### 2.5 Assessment of oxidative stress markers in liver

The liver kept at −20°C was used to measure oxidative stress enzymes. Liver tissue (1 g) was homogenized (1 min, 20 s homogenization with 5 s break) in 10 ml physiological serum (pH 7.4) at a ratio of 1:10 using a tissue homogenizer (Heidolph, Instruments Switzerland). The mixtures were transferred to a micro centrifuge (20,000 rpm for 10 min at 4°C). The supernatant was collected and used to measure reduced glutathione (GSH), total antioxidant capacity (TAC), Superoxide dismutase (SOD) activity, and malondialdehyde (MDA) concentration by using commercial kits (Arsam Farazist kits, Urmia, Iran). Total protein in the homogenate was measured by the method described by Lowry et al. (1951) using bovine serum albumin as control and enzyme activity was expressed as unit per mg protein (U/mg protein). GSH was measured using the thiol concentration (yellow) in glutathione which reacts with the element reagent namely dinitrothiocyanatebenzene (DNTB) and produces nitro thiobenzoate (TNB). The produced yellow color was quantified at 412 nm. The color intensity was directly proportional to the reductive thiol. Finally, the amount of GSH in mmol/mg of protein was expressed. The ABTS method was used to evaluate TAC. In this method, ABTS is oxidized by an oxidant and converted to ABTS·^+^ green which is inhibited in the presence of an antioxidant. The produced color is read at 414 nm. SOD activity was measured by the method of Sun et al. (1988). The activity of SOD is directly correlated with the degree of oxidation inhibition (Nitroblue tetrazolium) by the oxygen anion. The absorbance was read at 580 nm, and the enzyme activity was expressed as a unit in mg of protein. Lipid peroxidation in the liver tissue was estimated by measuring thiobarbituric acid reactive substances (TBAR_S_). The TBAR_S_ test is a quantitative direct test for measuring MDA. The rate of MDA was expressed in MDA nmol/mg of protein.

### 2.6 Liver tissue preparation and analysis

The liver tissues fixed in 10% formalin solution were used to find any abnormalities due to high‐fat diet feeding. After the process of fixation, drying, tissue passage, paraffin blocks, and 5‐microns sections were prepared. Then, these thin sections were stained with Sudan Black B and hematoxylin & eosin to see any abnormality under a light microscope (Brunt et al., 1999).

### 2.7 Analysis of mRNA expression of autophagy genes by RT‐qPCR

At the end of the experiment, the part of liver tissue was removed and kept at −20 for RNA extraction for determination of expression levels of autophagy genes (Beclin 1, Atg7, LC3‐ ɪ, P62). Total RNA was extracted (TRIzol® reagents) according to the company protocol and its amount and quality were determined using a nanodrop spectrophotometer (260 nm). The RNA with quality of more than 1.8–2 was considered for cDNA synthesis. The process was followed by cDNA synthesis according to the manufacturer's protocol (Pars Toos, CAT: A101161 Iran) in a 20 ml reaction mixture containing 1 mg RNA, oligo primer (1 μl), a buffer (4 μl), RNAse inhibitor (1 μl), dNTP mixture 10 mM (2 μl), and M‐MuLV reverse transcriptase (1 μl). Quantitative RT‐PCR experiments for each sample were performed using MyGo PCR (USA) thermal mini‐cycler in three versions. qPCR reaction mixtures contained 0.5 μl of cDNA pattern, 10 μl of 2× SYBER GREEN master (High ROX, Noavarane Tebe Beynolmalal, Iran), and 0.5 μl of forward and reverse primers of target genes (Table 2). Special primers were designed and produced by Gene Fanavaran Co. (Tehran, Iran) using a “Multiple alignment program for amino acid or nucleotide sequences” (MAFFT, V.7) (https://mafft.cbrc.jp/alignment/server). The primer pair sequences for each gene are presented in Table 2. The qPCR thermal cycling conditions were as follows: a general denaturation process at 95°C for 5 min followed by 40 denaturation cycles at 95°C for 20 s, annealing at 72°C for 30 s. Mean values of cycle threshold (CT) from triple readings of each gene were normalized considering the mean CT values of the internal control gene (GAPDH) and the relative expression levels of aforementioned genes were calculated using the ΔCt method: 2^−(dCt gene of interest − dCt internal control gene)^ (Gharehbagh et al., 2021).

**TABLE 2:** Nucleotide sequence of primers used for PCR

Gene	Primer	AT	Bp
P62	F: GCTGCTCTCTTCAGGCTTACAG	53°C	22
	R: CCTGCTTCACAGTAGACGAAAG		
Beclin‐1	F: AGCACGCCATGTATAGCAAAGA	51°C	22
	R: GGAAGAGGGAAAGGACAGCAT		
Atg7	F: AGCCTGTTCATCCAAAGTTCT	46°C	21
	R: CTGTGGTTGCTCAGACGGT		
LC3‐I	F: GATGTCCGACTTATTCGAGAGC	46°C	22
	R: TTGAGCTGTAAGCGCCTTCTA		
GAPDH	F: AAGGTCATCCATGACAACTT	58°C	20
	R: GGCCATCCACAGTCTTCTGG		

### 2.8 Statistical analysis

All data are expressed as mean ± standard division (SD) for *n* = 6 rats per treatment. The values were examined for normality (Shapiro–Wilks test) and homogeneity of variance (Levene's test), then one‐way ANOVA and Kruskal–Wallis tests were used to compare the means. Excel 2013 was used to draw the graphs and Spss software V.21 was applied to examine statistical changes in experimental treatments.

## 3 RESULTS

### 3.1 Effects of GT oligosaccharides on body and liver weight

The results of the effects of GT oligosaccharides on weight gain and relative weight of liver are presented in Table 3. The data revealed that the experimental diets affected on weight gain and the highest values were observed in the control which differed with the other groups (*p* < .05). There was no significant difference between HFD and GT oligosaccharides treatments (*p* > .05). Also, the results showed that the rats fed on HFD had higher relative weight of liver when compared with the other treatments (*p* < .05). No significant difference was observed among other groups (*p* > .05).

**TABLE 3:** The effects of GT oligosaccharides on weight gain and relative weight of liver in NAFLD‐induced rats

	Control	HFD	HFD + O100	HFD + O200
Weight gain (g)	63.6 ± 3.2^a^	56.6 ± 1.4^b^	56.5 ± 4.7^b^	51.6 ± 3.6^b^
Relative weight of liver	2.4 ± 0.1^b^	2.9 ± 0.08^a^	2.4 ± 0.18^b^	2.5 ± 0.18^b^

*Note*: Data are expressed as mean ± SD (*n* = 6 per treatment).

Different letters within a row indicate significant differences (*p* < .05).

Control (standard diet); high‐fat diet (HFD), high‐fat diet + 100 mg oligosaccharide/kg body weight (HFD + O100), high‐fat diet + 200 mg oligosaccharide/kg body weight (HFD + O200).

Relative weight of liver = [liver weight/body weight] × 100.

### 3.2 Effects of GT oligosaccharides on glucose regulation, insulin and HOMA‐IR index

**FIGURE 2:**
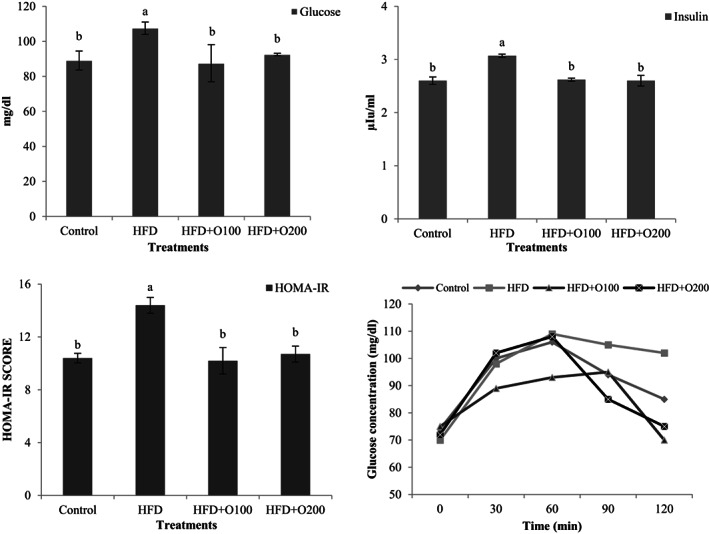
Fasting glucose, insulin, glucose tolerance, and HOMA‐IR values in the rats fed on HFD and TG oligosaccharides after 70 days experiment period. Data are presented as the mean ± SD (*n* = 6 per treatments). Results were statistically analyzed using one‐way ANOVA followed by Duncan's multiple‐comparison test, and values with different labels (a–c) are significantly different (*p* < .05). Control: Standard diet, HFD, High‐fat diet; HFD + O100, High‐fat diet + 100 mg/kg TG oligosaccharides per body weight of rat; HFD + O200, High‐fat diet + 200 mg/kg of TG oligosaccharides per body weight of rat.

Plasma concentrations of fasting glucose, insulin, glucose tolerance, and HOMA‐IR are presented in Figure 2. The results indicated that the rats fed on HFD had the highest plasma fasting glucose, insulin, and HOMA‐IR index in comparison with the other treatments (*p* < .05). There was no significant difference between control and GT oligosaccharides groups in aforementioned parameters (*p* > .05). In contrast to HFD treatment, GT oligosaccharide groups decreased glucose tolerance and the lowest tolerance was observed in aforementioned groups.

### 3.3 Liver function markers and plasma parameters

**FIGURE 3:**
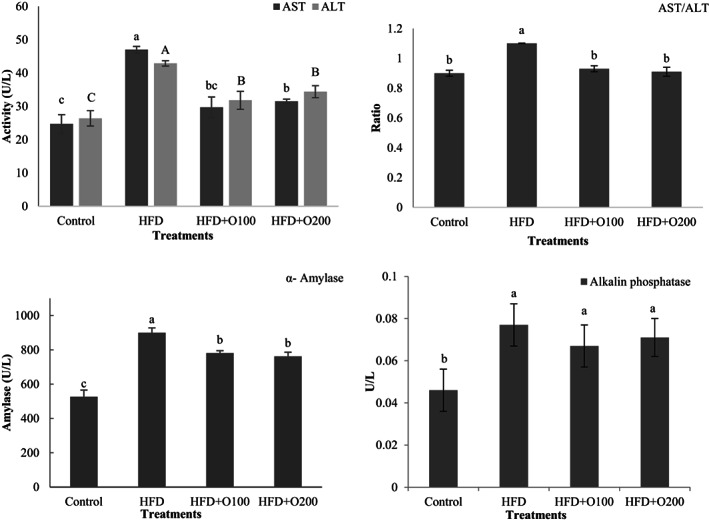
Effects of TG oligosaccharides on liver function markers and amylase, alkaline phosphatase activity in plasma of NAFLD induced rats. Data are presented as the mean ± SD (*n* = 6 per treatments). Results were statistically analyzed using one‐way ANOVA followed by Duncan's multiple‐comparison test, and values with different labels (a–c) are significantly different (*p* < .05). Control, Standard diet; HFD, High‐fat diet; HFD + O100, High‐fat diet + 100 mg/kg TG oligosaccharides per body weight of rat; HFD + O200, High‐fat diet + 200 mg/kg of TG oligosaccharides per body weight of rat.

The results of the effects of experimental diets on liver function markers (ALT, AST) are presented in Figure 3. Based on these results, the rats fed on HFD showed the higher values of AST and ALT in comparison with the other treatments (*p* < .05). Also, the results indicated that the use of GT oligosaccharides decreased significantly aforementioned markers when compared with the HFD group (*p* < .05). The lowest values for ALT and AST were observed in the control which differed with HFD and HFD + O200 treatments (*p* < .05).

Amylase and alkaline phosphatase were evaluated in plasma and the data are presented in Figure 3. The findings showed that there was significant difference between control, HFD and GT oligosaccharides (*p* < .05) and the lowest activity of amylase was observed in control, GT oligosaccharides and HFD respectively. A significant higher alkaline phosphatase activity was seen in HFD and GT oligosaccharides in comparison with control (*p* < .05). No significant difference was seen between the HFD and GT oligosaccharides (*p* > .05), although the highest activity was observed in HFD.

Plasma concentration of total triglyceride was significantly lower in HFD + O100 when compared with the control and HFD (*p* < .05) (Table 4). The plasma LDL, LDL/HDL ratio and total cholesterol levels of the HFD were significantly higher than those fed the control (*p* < .05). Treatments with oligosaccharides significantly decreased aforementioned parameters levels than those fed on HFD (*p* < .05). Also, the rats fed on oligosaccharide groups showed a significant higher HDL when compared with HFD treatment (*p* < .05).

**TABLE 4:** Effects of GT oligosaccharides on serum biochemical parameters in experimental treatments

	Control	HFD	HFD + O100	HFD + O200
Total triglyceride (mmol/L)	9.1 ± 0.4^ab^	10.5 ± 0.4^a^	6.8 ± 0.9^c^	7.1 ± 0.43^bc^
Total cholesterol (mmol/L)	2.5 ± 0.1^c^	3.4 ± 0.1^a^	3 ± 0.08^b^	3 ± 0.09^b^
HDL (mmol/L)	3.0 ± 0.05^bc^	2.8 ± 0.03^c^	3.3 ± 0.14^ab^	3.3 ± 0.03^a^
LDL (mmol/L)	0.59 ± 0.0^c^	1.1 ± 0.08^a^	0.84 ± 0.05^b^	0.8 ± 0.04^b^
LDL/HDL	0.19 ± 0.01^c^	0.4 ± 0.03^a^	0.26 ± 0.02^b^	0.23 ± 0.02^bc^

*Note*: Data are expressed as mean ± SD (*n* = 6 per treatment).

Different letters within a row indicate significant differences (*p* < .05).

Control (standard diet); high‐fat diet (HFD), high‐fat diet + 100 mg oligosaccharide/kg body weight (HFD + O100), high‐fat diet + 200 mg oligosaccharide/kg body weight (HFD + O200).

**FIGURE 4:**
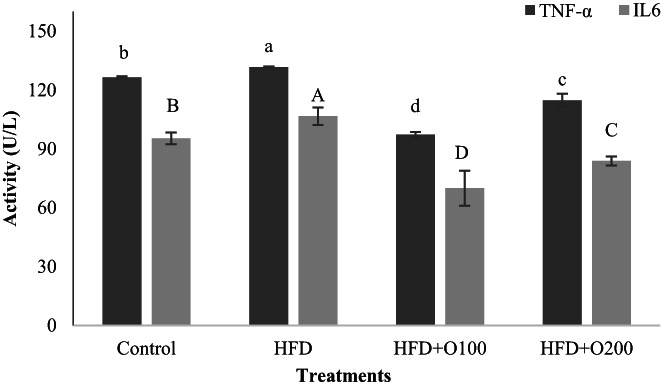
Effects of TG oligosaccharides on pro‐inflammatory factors in plasma of NAFLD induced rats. Data are presented as the mean ± SD (*n* = 6 per treatments). Results were statistically analyzed using one‐way ANOVA followed by Duncan's multiple‐comparison test, and values with different labels (a–c) are significantly different (*p* < .05). Control, Standard diet; HFD, High‐fat diet; HFD + O100, High‐fat diet + 100 mg/kg TG oligosaccharides per body weight of rat; HFD + O200, High‐fat diet + 200 mg/kg of TG oligosaccharides per body weight of rat.

The results of the effects of GT oligosaccharides on plasma pro‐inflammatory cytokines are presented in Figure 4. Our results showed that the use of GT oligosaccharides affected on TNF‐ɑ and there is significant difference between experiment treatments (*p* < .05). The higher values of TNF‐ɑ was seen in HFD, control, HFD + O200 and HFD + O100, respectively. In addition, IL6 levels changed in experimental groups, and in contrast to HFD, both doses of GT oligosaccharides decreased significantly IL6 level and the lowest value was observed in HFD + O100 treatment (*p* < .05). Interestingly, the level of IL6 was significantly lower than the control (*p* < .05).

### 3.4 Effects of GT oligosaccharides on oxidative stress

**FIGURE 5:**
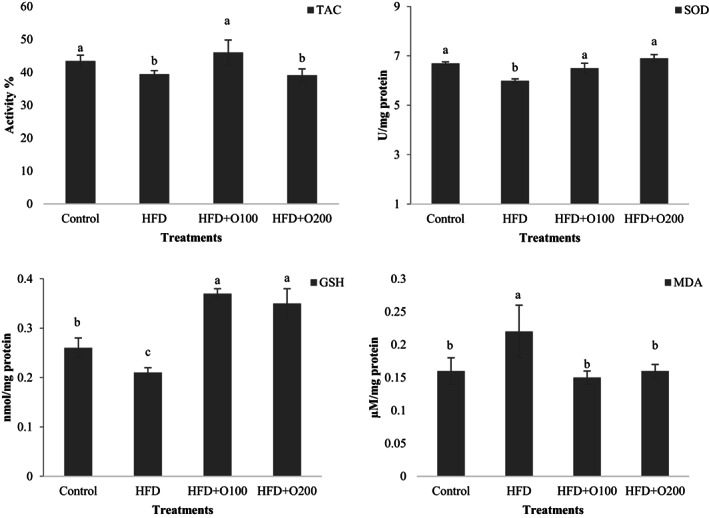
Effects of oral administration of TG oligosaccharides on the content of total antioxidant activity (TAC), GSH, SOD activity, and MDA concentration in liver tissue of the rats fed on experimental treatments. Data are presented as the mean ± SD (*n* = 6 per treatments). Results were statistically analyzed using one‐way ANOVA followed by Duncan's multiple‐comparison test, and values with different labels (a–c) are significantly different (*p* < .05). Control, Standard diet; HFD, High‐fat diet; HFD + O100, High‐fat diet + 100 mg/kg TG oligosaccharides per body weight of rat; HFD + O200, High‐fat diet + 200 mg/kg of TG oligosaccharides per body weight of rat.

At the end of the experiment, liver oxidative status was evaluated in NAFLD induced rats and the results are presented in Figure 5. The data indicated that the use of the GT oligosaccharides affected on the TAC and the highest values were seen in the control and HFD + O100 groups which differed with the others (*p* < .05). Also, the rats fed on the HFD and HFD + O200 treatments showed low level of TAC and no significant difference was seen between later groups (*p* > .05). A significant lower SOD activity was observed HFD treatment which differed with the others (*p* < .05). No significant difference was seen between control and GT oligosaccharides (*p* > .05). The highest GSH values were observed in GT oligosaccharides groups when compared with control and HFD treatments (*p* < .05). The lowest GSH was seen in HFD and control, respectively, which had significant different (*p* < .05). The concentration of MDA was increased significantly when the rats were fed HFD treatment as compared with the others (*p* < .05). No significant difference was seen between control and GT oligosaccharides (*p* > .05).

### 3.5 Effects of GT oligosaccharides on expression of liver autophagy genes

**FIGURE 6:**
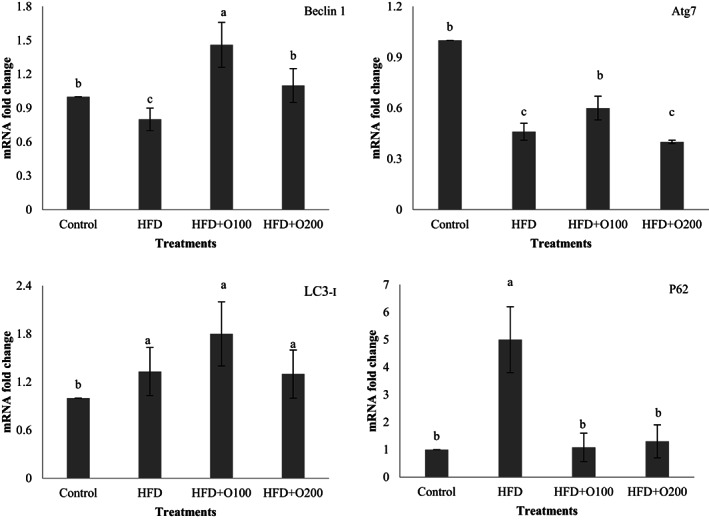
Expression of autophagy genes (Beclin 1, Atg7, LC3‐ɪ, and P62) in the liver of the induced NAFLD rats. Data are presented as the mean ± SD (*n* = 6 per treatments). Results were statistically analyzed using one‐way ANOVA followed by Duncan's multiple‐comparison test, and values with different labels (a–c) are significantly different (*p* < .05). Control, Standard diet; HFD, High‐fat diet, HFD + O100, High‐fat diet + 100 mg/kg TG oligosaccharides per body weight of rat; HFD + O200, High‐fat diet + 200 mg/kg of TG oligosaccharides per body weight of rat.

To determine the effects of HFD on liver autophagy, the alteration of Beclin 1, Atg7, LC3‐ɪ, and P62 expression was measured in the rat's liver (Figure 6). At the end of the experiment, the results showed that the animals fed on HFD showed significantly lower Beclin 1 expression when compared with other treatments (*p* < .05). The highest expression for Beclin 1 was seen in HFD + O100 which differed significantly with control and HFD + O200 (*p* < .05). Based on these results, feeding on HFD, and HFD + O200 treatments significantly decreased Atg7 expression while the rats fed on HFD + O100 showed higher expression (*p* < .05). Dietary treatments significantly affected on the expression of LC3‐ɪ and the highest values were observed in HFD and GT oligosaccharides treatments when compared with control (*p* < .05). No significant difference was observed in HFD and GT oligosaccharides treatments (*p* > .05), although higher value was seen in HFD + O100 treatment. Also, the rats fed on HFD showed significantly higher expression of P62 among experimental treatments (*p* < .05). No significant difference was observed between GT oligosaccharides and control (*p* > .05).

## 4 DISCUSSION

In the past few years, dietary natural compounds have gained a great attention due to the potential to improve NAFLD (Pan et al., 2014; Tao et al., 2019). Gum tragacanth and its natural oligomers shown beneficial effects, including antioxidant, antitumor, anti‐inflammatory, antimicrobial, anti‐hypertensive, anti‐obesity, and prebiotic activities (Gavlighi et al., 2013; Pirbalouti & Imaniyan‐Fard, 2016). In the current study, the results indicated that GT oligosaccharides (especially at a low dose of 100 mg/kg BW) positively affected on HFD induced rats and regulated glucose metabolism and ameliorate liver lipid accumulation. Also, we identified a beneficial effect of GT oligosaccharides on decreasing pro‐inflammatory factors and oxidative stress, so as to alleviate liver injury and steatosis in the rats.

The results of present study indicated that experimental diets affected on weight gain and the lowest values were observed in HFD and HFD + TG treatments. It seems that palatability and high energy content of HFD groups affected on food intake and consequently weight loss in the rats (Nasri et al., 2015). In contrast to control and GT oligosaccharides, relative weight of liver increased in HFD. Black Sudan and H&E staining of liver tissue also demonstrated the beneficial effects of GT oligosaccharides against HFD‐induced hepatic lipid accumulation. Based on these results, compared to those in control and GT oligosaccharides treatments, lipid droplets and lipid vacuoles were increased in HFD, whereas in the GT oligosaccharides groups, lipid accumulation was decreased by alleviating histological changes.

**FIGURE 7:**
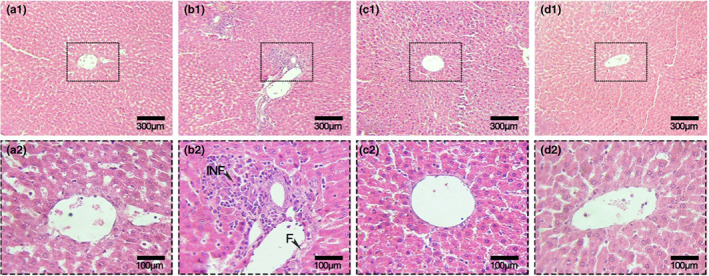
Hematoxylin and eosin (H&E) staining of liver tissue sections from control (A1, A2), HFD (B1, B2), HFD + O100 (C1, C2), HFD + O200 (D1, D2) (×400). F, Cell fibrosis; INF, Infiltration.

**FIGURE 8:**
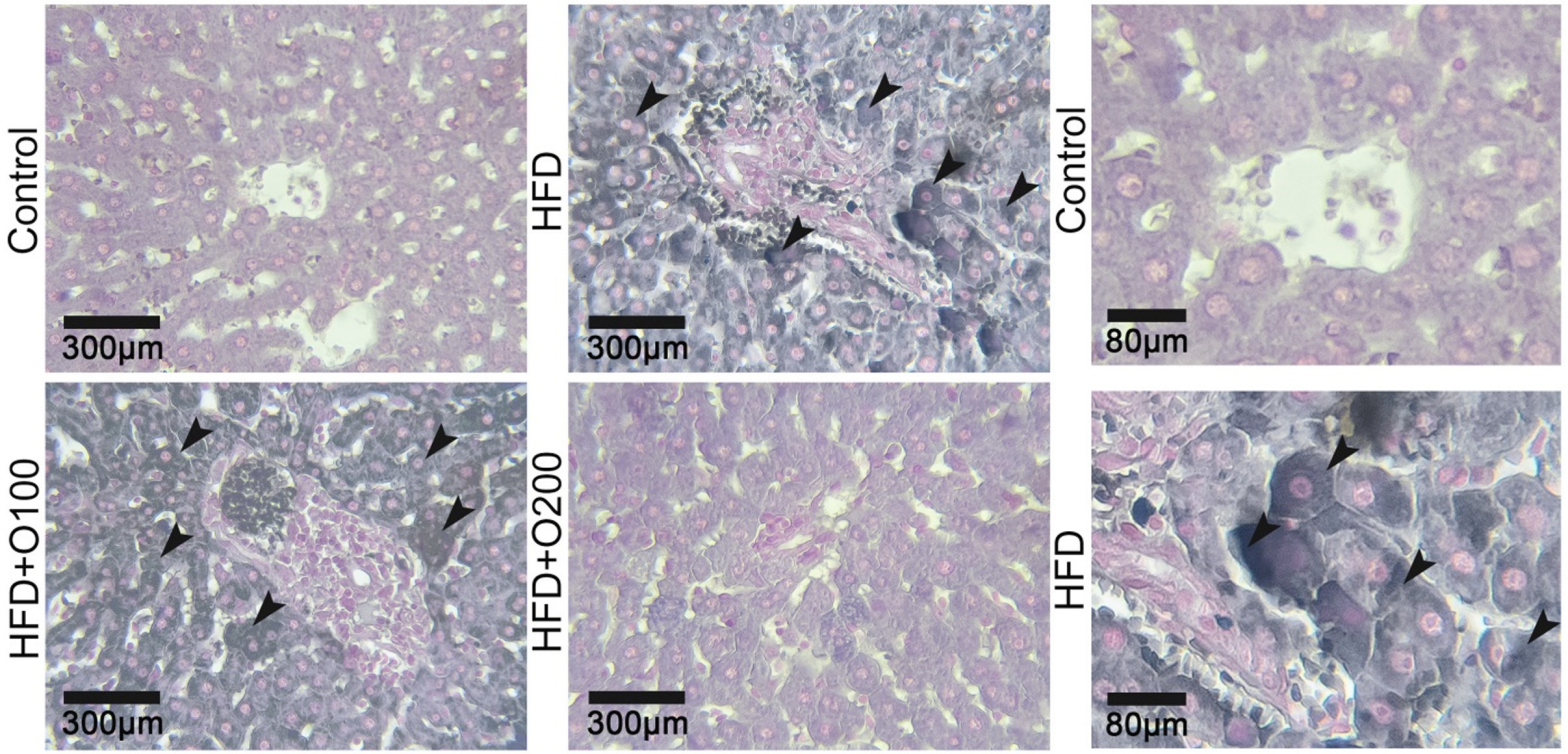
Sudan black staining of liver tissue sections from control, HFD, HFD + O100, HFD + O200.

In the current study, the rats fed on HFD resulted in an increase of plasma total triglyceride and hepatocytes lipid accumulation (Figures 7 and 8). The increase in relative weight of liver in Table 3 validates these results. In contrast, the use of GT oligosaccharides in the experimental diets significantly decreased plasma total triglyceride, cholesterol, LDL, and LDL/HDL ratio. Interestingly, the rats fed on GT oligosaccharides(HFD + O100, HFD + O200) increased plasma HDL concentration and it seems that the GT oligosaccharides regulate HDL concentration by lipid catabolism, as HDL can transport cholesterol from peripheral tissues to the liver for oxidation (Muanprasat & Chatsudthipong, 2017; Tao et al., 2019). In accordance with decreased total triglycerides, cholesterol, and reduced liver weight owing to GT oligosaccharides, the H&E and black Sudan staining histological analyzes demonstrated the protective effects of GT oligosaccharides on NAFLD.

Moreover, our findings indicated that the rats fed HFD showed significantly higher plasma concentration of ALT and AST compared to the control and GT oligosaccharides groups. ALT and AST are known an important biomarkers related to hepatocytes function, and the increased plasma concentration of aforementioned enzymes implied occurrence of Cholestasis, imbalance of lipid and carbohydrate metabolism, hepatotoxicity and hepatocytes injury, which are associated with NAFLD and hyperlipidemia (Chang et al., 2013; Khodadoostan et al., 2016; Taliercio et al., 2013). Our finding showed that the using of GT oligosaccharides lower HFD induced high plasma concentration of ALT and AST, indicating that administration of GT oligosaccharides protect our rats against HFD induced liver damage.

Oxidative stress occurs in several organs and causes the development of metabolic syndrome in NAFLD rats (Grundy, 2004). Matsuzawa et al. (2007) reported that accumulation of cholesterol and triglycerides induced by HFD accelerates oxidative stress in the hepatocytes. Different processes can cause the creation of reactive oxygen species (ROS), which can lead to oxidative damage and degradation of the antioxidant system. Two main pathways for the production of ROS have been reported in NAFLD (Chitapanarux et al., 2009; da Cunha et al., 2014). Lipid accumulation and high consumption of oxygen by the mitochondrial respiratory chain, which occurs due to oxidative phosphorylation. High mitochondrial respiratory chain caused by lipid oxidation leading to MDA synthesis as one of the tissue damage markers in the liver (Marnett, 1999). In the present study, feeding the rats on HFD significantly increased MDA, which is in line with other results that show HFD increases oxidative indicators (Mamun et al., 2019; Rahman et al., 2017). As the amount of MDA increases, the activity of other antioxidant markers like TAC, SOD activity, and GSH level decreases due to a sharp increase in free radicals in liver cells. The beneficial effects of GT oligosaccharides on oxidative stress obtained in the current study where MDA concentration decreased and TAC, SOD activity as well as GSH levels increased. It seems that GT oligosaccharides reduce stress levels by using various mechanisms including upregulation of antioxidant enzymes genes (Tao et al., 2019), production of stable products with electron donor (Zou et al., 2016), and free radical scavenging (Zou et al., 2013).

Pro‐inflammatory response in hepatic steatosis may be promoted by various stimuli, such as liver enzymes (ALT, AST), oxidative stress, total cholesterol, and triglycerides, and is characterized by increases of TNF‐ɑ and IL‐6 in the liver (Kazankov et al., 2019; Kim et al., 2010). An increase in oxidative stress and liver enzymes stimulate pro‐inflammatory cytokines in the bloodstream, resulting in reduced lipid degradation and hepatic lipid overloading and thus, they produce direct influence on pathogenesis of NAFLD (Carter‐Kent et al., 2008; Mao et al., 2016). The results of previous studies showed that treating the mice with anti‐TNF antibodies improved NAFLD, indicating that the drugs, which can prevent inflammatory response, may be beneficial for NAFLD (Li et al., 2003). In our study, the results shown that HFD stimulates pro‐inflammatory cytokines while the use of GT oligosaccharides suppressed secretion of TNF‐ɑ and IL‐6 which indicates beneficial for NAFLD.

Previous studies reported that the high‐fat diets change gut microbial community and decrease lactobacilli (beneficial microbial genera) and lead to increased metabolic syndrome (Daniel et al., 2014; Singh et al., 2016). Experiments on the human and animal models have shown that probiotics effectively decrease colon inflammation, hepatic steatosis, insulin resistance, and glucose concentration (Festi et al., 2014; Wang et al., 2015). To the best of our knowledge, oligosaccharides are known as prebiotics which have been exhibited to stimulate health promoting bacteria (Ehara et al., 2016). In previous studies, the prebiotics potential of GT oligosaccharides has recently been addressed and the findings showed that GT oligosaccharides stimulate growth of beneficial bacteria like bifidobacteria strains and lactobacillus spp. (Gavlighi et al., 2013). In our study, in contrast to HFD, the use of GT oligosaccharides improved fasting glucose, insulin, and glucose tolerance in NAFLD‐induced rats. The mechanisms underlying the anti‐hyperglycemic activity of oligosaccharides include the protection of pancreatic cells, and the promotion of glucose uptake, insulin secretion, the alleviation of insulin resistance, inhibition of carbohydrate‐hydrolyzing enzymes, and the improvement of gut microbiota dysbiosis (Tao et al., 2021).

Autophagy is an important intracellular mechanism in the homeostasis of cells and their long‐term survival (Gharehbagh et al., 2021). This mechanism controls the amount and quality of cytoplasmic contents in eukaryotic cells, which includes digestion of damaged proteins and organelles, lipids, and carbohydrates (Singh et al., 2009; Yang et al., 2010). A set of factors such as oxidative stress, chronic inflammatory and lipotoxicity as well response upon autophagy suppression often increase hepatocyte cell death (Madrigal‐Matute & Cuervo, 2016). In selecting medicines, efforts should be made to increase liver autophagy that may reduce the progression of liver diseases involving inflammation or injury, including non‐alcoholic fatty liver (NAFLD) or non‐alcoholic steatohepatitis (NASH) (Czaja, 2010). Among the genes involved in autophagy, Beclin‐1, Atg7, LC3‐ɪ, and P62 genes play a major role in this process. Beclin‐1 Builds the primary autophagosomal nucleus in this process, Atg7 and LC3‐ɪ complete the autophagosome wall with the help of some proteins (Ryter et al., 2014). Gene P62 is the main and influential gene in the autophagy and transfers the damaged organelles and lipid droplets to autophagosomes for decomposition and completes the autophagy process (Liu et al., 2016). In this study, the expression of the aforementioned genes was investigated, for which the results are presented in Figure 6. Based on the results, the lowest expression of Beclin‐1 and Atg7 genes was observed in HFD treatment, indicating oxidative conditions, lipid accumulation, and lack of homeostasis inside the hepatocytes. The findings were confirmed by the results of antioxidant status, liver enzymes (ALT, AST), histopathological observations, and the presence of inflammatory factors in the treatment. Furthermore, the use of GT oligosaccharides, especially at low concentrations significantly increased the expression rates of Beclin‐1 and Atg7 genes. Interestingly, the highest expression of LC3‐ɪ was seen in HFD and GT oligosaccharides treatments than the control. Koga et al. (2010) and Gonzalez‐Rodriguez et al. (2014) reported that lipid changes in autophagosome membranes and decrease vesicles' ability to connect with lysosomes, resulting in reduction in autophagy process. This reduction in autophagosome clearance could explain the accumulation of LC3‐II and p62 observed in NAFL and NASH patients and is positively related to disease severity (Gonzalez‐Rodriguez et al., 2014; Koga et al., 2010).

## 5 CONCLUSION

In conclusion, the current study demonstrated that GT oligosaccharides extracted from Gum tragacanth (*Astragalus gossypinus*) exerted protective effects against NAFLD in HFD induced rats. Based on our results, GT oligosaccharides improved oxidative status, liver enzymes, and pro‐inflammatory factors in the rats fed on the HFD. Also, the results indicated that GT oligosaccharides regulate fasting glucose, postprandial glucose, and insulin concentration in NAFLD induced rats. In addition, GT oligosaccharides at low concentration stimulate expression of autophagy genes and help the liver cells to make homeostasis and their long‐term survival. Based on these results and nutritional properties, GT oligosaccharides could be used as a useful nutritional strategy in NAFLD treatment and prevention.

## FUNDING INFORMATION

This study was supported by the office of vice chancellor for research at Urmia University (Thesis No. 4542).

## CONFLICT OF INTEREST

There is no conflict of interest in this paper.

## Data Availability

The data used in this paper are available in case of reviewer or editor request.
