# Erratum to “Low‐molecular weight oligosaccharides from gum tragacanth (*Astragalus gossypinus*) ameliorate nonalcoholic fatty liver disease (NAFLD) in Wistar male rats”

**DOI:** 10.1002/fsn3.3349

**Published:** 2023-04-13

Hossein Zadeh, Z., Najdegerami, E. H., Niko, M., Nejati, V., & Ahmadi Gavlighi, H. (2023). Low‐molecular weight oligosaccharides from gum tragacanth (*Astragalus gossypinus*) ameliorate nonalcoholic fatty liver disease (NAFLD) in Wistar male rats. *Food Science & Nutrition*, 11, 765–777. https://doi.org/10.1002/fsn3.3112

In the originally published version of the article, the third author's name was erroneously misspelled as Mehdi Niko. The spelling has now been corrected to Mehdi Nikoo.

